# CD34 Expression in the Stromal Cells of Alveolar Adenoma

**DOI:** 10.1155/2012/913517

**Published:** 2012-10-16

**Authors:** Nicolina De Rosa, Alfonso Maiorino, Ilaria De Rosa, Carlo Curcio, Carmine Sellitto, Dario Amore

**Affiliations:** ^1^Complex Operative Unit of Pathology, AORN Monaldi, Naples, 80131 Naples, Italy; ^2^Complex Operative Unit of Thoracic Surgery, AORN Monaldi, Naples, Italy; ^3^School of Medicine, Second University of Naples, Italy

## Abstract

The alveolar adenoma of the lung is a rare benign tumor characterized by a proliferation of both the alveolar epithelial cells and the mesenchymal septal cells. Immunohistochemically, the epithelial cells stain for cytokeratin (CK) AE1AE3, CK7, thyroid transcription factor 1 (TTF1), and surfactant apoprotein confirming the derivation by the type 2 pneumocytes. The stromal cells are negative for these markers but they show focally smooth muscle and muscle-specific actin positivity. We describe two cases that showed immunohistochemically a CD34 positivity of the mesenchymal septal cells. This aspect has been previously described in a two cases report, but not emphasized by the authors as a distinctive feature of the lesion. We consider this CD34 positivity as a marker of immaturity or stemness of the lesional septal spindle cells, that could be responsible of the different phenotypic and morphological profile of the interstitial cells, that could be, therefore, considered neoplastic and not reactive.

## 1. Introduction 

Alveolar adenomas are rare benign peripheral lung tumors first described by Yousem and Hochholzer [1]. Only a few have been reported since [2–12] This neoplasm presents as a solitary peripheral lesion discovered incidentally on chest radiographs.

Macroscopically these tumours manifest as well-demarcated spongy nodules with average size of 1.9 cm, which may be located in any lobe beneath an intact pleura.

Microscopically, they possess unique histological characteristics, which allow diagnosis by light microscopy alone: the cystic spaces dominate the picture, with the larger cysts usually concentrated in the middle of the tumour. The alveolar lumina contain few histiocytes, erythrocytes, and an eosinophilic proteinaceous granular material. The cysts are lined with a single layer of epithelial cells, with most of them being cuboidal or “hob nailed” in appearance and eosinophilic, finely vacuolated, or foamy cytoplasm, consisting in type 2 pneumocytes as showed by immunohistochemical and ultrastructural studies [1, 5, 6]. 

The interstitial component varies from a thin connective tissue layer resembling normal alveolar septa to markedly thickened alveolar walls with prominent spindle/oval-shaped cells containing a few mixed macrophages, plasma cells, and lymphocytes.

The prognosis is favourable and conservative surgical excision is curative.

## 2. Cases Reports

### 2.1. Clinical Findigs

**Case 1**

A 24-year-old, nonsmoker man underwent a chest X-ray, during a clinical screening before employment in the army, that occasionally revealed a solitary peripheral nodule in the lower lobe of the left lung.

A physical examination was unremarkable.

A thoracic computed tomography (CT) scan showed a well circumscribed homogeneous noncalcified mass of 18 × 17 mm. with contrast enhancement in intranodular areas, with no other abnormal finding.

Although a positron emission tomography (PET) scan revealed the benign nature of the lesion, a thoracoscopic wedge resection was performed.

Seven months after tumour resection the patient is alive and well, without recurrent disease.

**Case 2**

A 35-years-old, nonsmoker woman presented with complaints of right sided pleuritic chest pain of a few weeks duration. A chest roentgenogram revealed a right upper lobe nodule approximately 5 cm in diameter. A computerised tomography (CT) scan verified the finding of a well-circumscribed pleural-based right upper lobe nodule, with no other abnormal finding.

Because of the patient's continuous symptoms and concern for possible malignancy, the nodule was surgically removed. The patient underwent thoracotomic biopsy with a wedge resection of the lesion in the right upper lobe.

The patient 11 years later is alive and there are no signs of recurrence.

## 3. Material and Methods

Pulmonary biopsies were fixed in 10% neutral buffered formalin and paraffin embedded. Hematoxylin and eosin stained sections were performed for light microscopy. 

Additional sections for the immunohistochemical study were obtained, utilizing antibodies against cytokeratins CKAE1/3, CK 7, CK20, CK5/6, CKHMW, vimentin, S100 protein, epithelial membrane antigen (EMA), neuron-specific enolase (NSE), factor VIII, desmin, specific muscle actin (HHF35), smooth muscle actin (1A4), CD31, CD34, thyroid transcription factor-1 (TTF1), CD56, CD57, surfactant protein A, chromogranin A, and synaptophysin. All the antibodies were prediluted and supplied by Ventana. The determinations were performed by the Ventana Bench-Mark XT Autostainer following the manufacturer instructions. 

## 4. Result 

### 4.1. Pathologic Findings

**Case 1**

**Figure 1:**
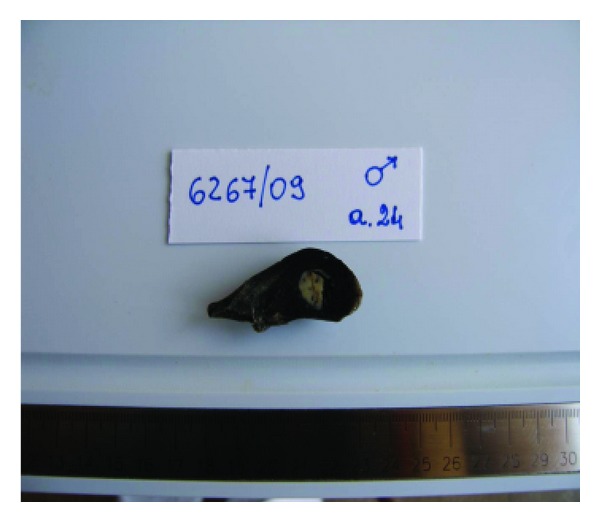
The resected specimen showed a small and well-defined solid nodule.

The resected lung subsegment contained a well-defined nodular lesion measuring 18 mm of maximum diameter. The cut surface was solid with peripheral fissure with a homogeneous grey appearance. The remaining lung tissue was unremarkable (Figure 1).

**Figure 2:**
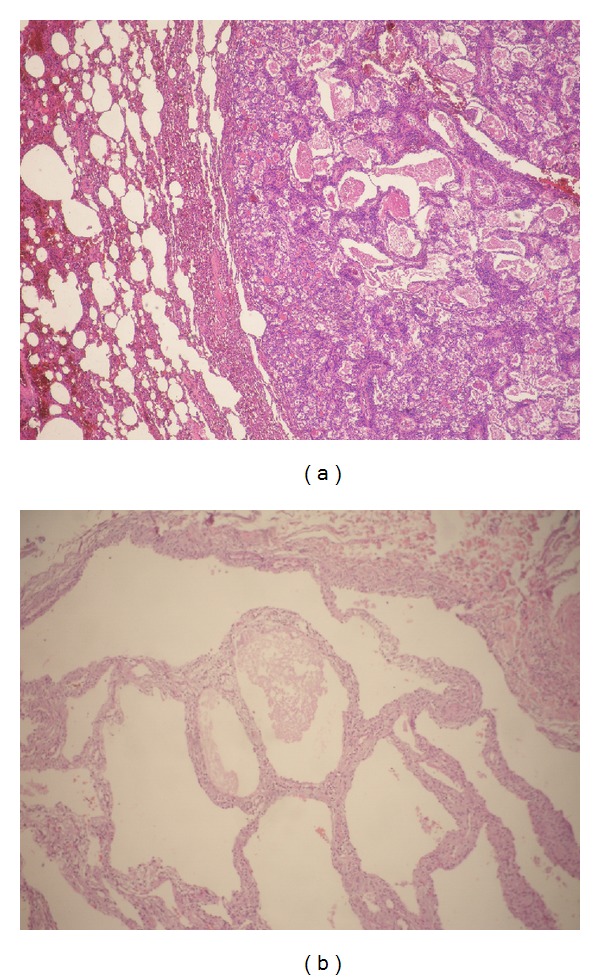
(a) low magnification showing sharp demarcation from the adjacent lung parenchyma and cystic spaces larger in the central area of the tumour. (b) the alveolus-like spaces of this tumor are filled with a finely granular proteinaceous material and thin walls (case 1).

On light microscopy the tumour was well demarcated and consisted of multiple cystic spaces of variable size, larger in the central areas, and smaller at the periphery of the lesion. The spaces were lined by plump cells with a hobnail appearance and contained intraluminal granular proteinaceous debris and foamy or vacuolated alveolar macrophages (Figure 2(a)). The intervening septa were thick and composed of spindle cells and delicate collagen fibres. Furthermore, they were richly vascularized with numerous vessels ranging in size from small-to-large ectatic capillaries. Focally, aggregates of lymphocytes were present. Foci of interstitial haemorrhage and haemosiderosis were also observed.

Mitotic activity was inconspicous and no atypical features were noted. 

Immunohistochemically, the cells lining the cystic spaces were reactive with antibodies against various cytokeratin (CK AE1/3, CK7), EMA, TTF1, and surfactant apoprotein. 

**Figure 3:**
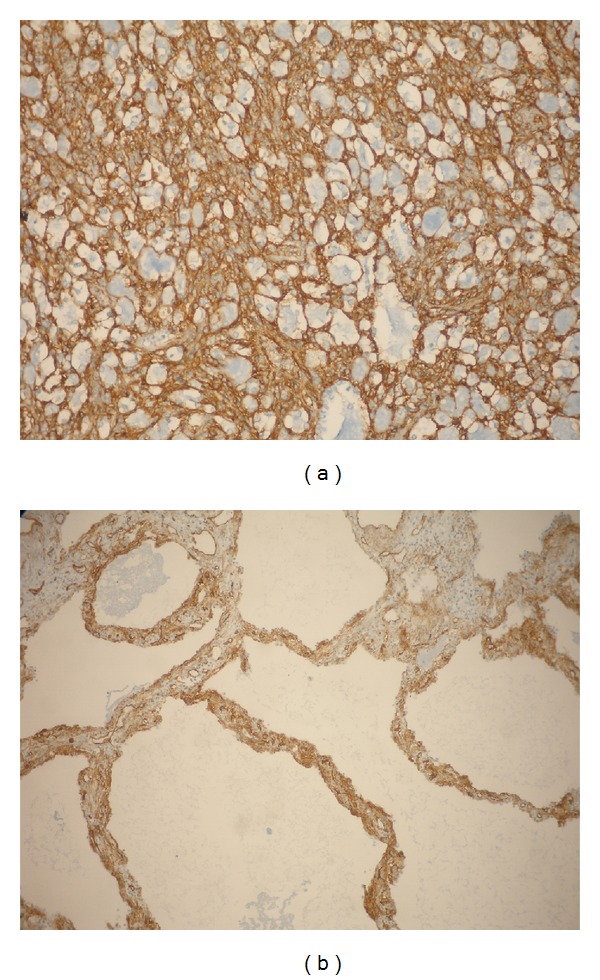
(a) CD34 positivity in the stromal cells (case 2). (b) CD34 positivity in the stromal cells (case 2).

The interstitial spindle cell component reacted with vimentin and CD34 diffusely and intensely (Figure 3(a)); only sporadic cell reacted with muscle-specific actin.

**Case 2**

Macroscopically, the specimen appeared well demarcated, multicystic and measured 5 cm in diameter. The cysts had a thin, translucent walls. It was reminiscent of peritoneal multicystic mesotheliomas.

Microscopically, the proliferation was well demarcated from the adjacent, compressed lung parenchyma; the cysts containing amorphous granular material and they were lined by attenuated, flattened cytologically bland epithelium.

The cystic spaces were separated by delicate myxoid stroma with bland spindle or oval shaped mesenchymal cells and with mild inflammatory infiltrate (Figure 2(b)).

To the immunohistochemical study, the epithelial lining cells stained for CK AE1/3, CK7, EMA, TTF1, and surfactant apoprotein indicating a type II pneumocyte phenotype, while stromal cells were positive for vimentin, for CD34 (Figure 3(b)), more intensively in the subepithelial spindle cells and focally for muscle-specific actin.

A well developed, fine capillary network that traversed the interstitial mesenchyme was identified by vascular markers. 

## 5. Discussion

Alveolar adenoma of lung is an extremely rare benign neoplasm of uncertain histogenesis with distinctive gross and microscopic findings. 

Some authors have suggested that they may represent the benign counterpart of bronchioloalveolar carcinoma [4].

The exact number of reported cases is difficult to ascertain because these tumours are often confused with other rare lung tumours (case 1).

Radiologically, it usually presents of a solitary lung solid nodule; however, a case presenting with a cystic aspect has been reported [7] therefore, correct diagnosis requires accurate recognition of the characteristic histological features. 

Pathologically, the differential diagnosis includes lymphangioma, pneumocytoma, bronchioloalveolar carcinoma and metastatic microcystic low-grade stromal sarcomas of uterus.

Its histogenesis is poorly understood: in particular, it is not known whether both the epithelial and mesenchymal components are neoplastic. Cavazza et al. [7] have observed microsatellite alterations and LOH only in the epithelial component suggesting that these two components are genetically heterogeneous, but it is not understood what component (epithelial and/or mesenchymal) is neoplastic.

We think that the immunohistochemical CD34 positivity of the interstitial spindle cells is confirming the possible derivation by a primitive mesenchymal cell of the alveolar septum adjacent to the capillary constituting the “thick” nongas-exchanging portion of the septum. 

CD34 is a marker of adipose-derived stem/stromal/progenitor cells (ASCs) but its expression in human ASCs (hASCs) decreases over time in culture.

Suga et al. studied the different aspects of CD34+ and CD34− hASCs in culture. They found different biological properties; in fact CD34+ cells were more proliferative and were able to form colonies; conversely CD34− cells tended to differentiate into specific lineages such as adipose, bone, or smooth muscle [13].

These features could be the key to explain, therefore, the different results in immunophenotype of stromal cells of the alveolar adenoma, as well as could justify the unusual morphological adipose differentiation reported in a case by Cavazza et al. [7]. This latter finding, furthermore, makes possible the hypothesis of the neoplastic origin of the stromal spindle component.
